# 3D printed integrated bolus/headrest for radiation therapy for malignancies involving the posterior scalp and neck

**DOI:** 10.1186/s41205-022-00152-w

**Published:** 2022-07-18

**Authors:** Eric J. Hsu, David Parsons, Tsuicheng Chiu, Andrew R. Godley, David J. Sher, Dat T. Vo

**Affiliations:** 1 Department of Radiation Oncology, Division of Clinical Radiation Oncology, UT Southwestern Medical Center, Dallas, TX 75390 USA; 2 Department of Radiation Oncology, Division of Medical Physics and Engineering, UT Southwestern Medical Center, Dallas, TX 75390 USA

**Keywords:** 3D Printing, Bolus, Radiation therapy, Scalp malignancy, Neck Malignancy

## Abstract

**Background:**

Malignancies of the head and neck region, encompassing cutaneous, mucosal, and sarcomatous histologies, are complex entities to manage, comprising of coordination between surgery, radiation therapy, and systemic therapy. Malignancies of the posterior scalp are particular challenging to treat with radiation therapy, given its irregular contours and anatomy as well as the superficial location of the target volume. Bolus material is commonly used in radiation therapy to ensure that the dose to the skin and subcutaneous tissue is appropriate and adequate, accounting for the buildup effect of megavoltage photon treatment. The use of commercially available bolus material on the posterior scalp potentially creates air gaps between the bolus and posterior scalp.

**Case presentations:**

In this report, we created and utilized a custom 3D-printed integrated bolus and headrest for 5 patients to irradiate malignancies involving the posterior scalp, including those with cutaneous squamous cell carcinoma, melanoma, malignant peripheral nerve sheath tumor, and dermal sarcoma. Treatment setup was consistently reproducible, and patients tolerated treatment well without any unexpected adverse effects.

**Conclusions:**

We found that the use of this custom 3D-printed integrated bolus/headrest allowed for comfortable, consistent, and reproducible treatment set up while minimizing the risk of creating significant air gaps and should be considered in the radiotherapeutic management of patients with posterior scalp malignancies.

**Supplementary Information:**

The online version contains supplementary material available at 10.1186/s41205-022-00152-w.

## Background

Malignancies of the head and neck region, including cutaneous malignancies and sarcomas, are commonly managed with surgery. However, there is a proportion of patients with high-risk malignancies that can cause significant morbidity and mortality who require a multidisciplinary treatment approach including surgery, radiotherapy, and even consideration of systemic therapy [1]. Radiation therapy serves as an important modality for these malignancies in the definitive, adjuvant, or palliative settings.

To maximize treatment efficacy, it is of utmost importance to ensure that the radiation target properly covers the tumor or tumor bed to ensure adequate dosing. For skin cancers or sarcomas that are close to the skin, the radiation target includes the tumor bed, which can reside at or near the skin surface. The use of megavoltage photons typically has a skin-sparing effect as it requires dose buildup in the first superficial micrometers of the skin [2]. For this reason, the use of a bolus (also known as a tissue equivalent) can increase the dose at the skin surface by allowing the prescribed dose to build up in the bolus prior to approaching the skin.

Commercially available bolus material has a specific gravity close to water, typically around 1.02, and can be produced with materials such as polystyrene or synthetic gel. This commercially available bolus material is typically applied as a square sheet that is cut at the time of CT simulation to ensure adequate coverage of the skin target. However, the use of this commercially available planar bolus material can result in significant frequencies of air gaps of over 5 mm and increased risk of inadequate dosing of the superficial or cutaneous radiation targets, especially in areas of irregular surface contours, such as the head and neck, where a significant number of cutaneous malignancies may arise [3].

In our clinic, we have improved our clinical practice towards the use of 3D-printed bolus that is custom designed and implemented in patients to ensure improved dosimetry and workflow efficiency while reducing the day-to-day variation in set up and potential risk of inadequate or inappropriate skin dosing [4–6]. The use of the 3D-printed bolus allows for appropriate dosimetric planning for complex and irregularly shaped surfaces and targets, especially in the head and neck region where the use of a planar commercially available bolus may not conform to the surface without the creation of air gaps. In addition, there may be some anatomic locations where the placement of bolus material may be inaccessible, such as the posterior scalp and neck. Previous reports have proposed synthesizing custom 3D-printed boluses for scalp irradiation, but no reports have studied the application of this technology for irradiation of tumors in the posterior scalp [7, 8]. In this report, we describe five cases of the creation and implementation of a 3D-printed bolus that is integrated into a headrest for the radiation treatment of cutaneous malignancies located on the posterior scalp.

## Case presentations

### Patient case 1

The first patient is a 64-year-old male that initially presented with a lesion on his scalp that was biopsied with a pathology consistent with moderately differentiated squamous cell carcinoma. The patient’s history was unremarkable except for extensive prior sun exposure. Further work-up including CT of the neck and PET/CT showed a lesion located in the left parietal-occipital scalp with satellite nodules as well as multiple enlarged left cervical lymph nodes. The patient then underwent radical resection of the left posterior scalp tumor, outer calvarial bony resection, and left neck with application of Integra. The pathology was consistent with invasive adenosquamous carcinoma measuring 3.5 cm. The tumor was positive for extensive lymphatic spread but negative for perineural invasion. The margins were negative for carcinoma within the stroma but on the 6–9 o'clock margin, there was carcinoma within lymphatic spaces. There were also 13 lymph nodes identified with metastatic carcinoma in 75 examined lymph nodes. No extranodal extension was identified. The tumor was staged as T3N2b.

Due to the presence of extensive lymphatic spread and multiple lymph node involvement, adjuvant radiation therapy was recommended to the tumor bed located on the left posterior parietal scalp and left neck, with a recommended dose of 60 Gy delivered in 30 fractions. There were no positive margins or presence of extranodal extension, for which concurrent chemotherapy may be added, based on data from mucosal head and neck cancers, although the role of concurrent chemotherapy and optimum regimen is controversial in the management of cutaneous squamous cell carcinoma [9–12].

### Patient case 2

The second patient is a 90-year-old male with past medical history significant for chronic lymphocytic leukemia, currently undergoing observation, presenting with pleomorphic dermal sarcoma with involvement of the posterior scalp and bilateral neck.

The patient initially presented with a nodule located on the posterior scalp that was biopsied as a poorly differentiated malignant neoplasm, suspected to be squamous cell carcinoma. On physical examination, the patient had multiple areas of lymphadenopathy including the right occipital lymph node and right posterior cervical triangle lymph nodes. Fine-needle aspiration was performed on the lymph nodes which revealed a spindle and epithelioid malignant neoplasm. Imaging work-up, including diagnostic CT and PET/CT, showed bilateral posterior scalp nodules as well as bilateral cervical lymphadenopathy. Fine-needle aspiration of the posterior left occipital lymph node was consistent with pleomorphic dermal sarcoma. In addition, there was a right paratracheal lymph node that was fine-needle aspirated, which was also consistent with malignant neoplasm compatible with metastatic pleomorphic dermal sarcoma.

He underwent left posterior neck dissection with sacrifice of the trapezius, wide local excision of posterior scalp malignancy, right posterior lateral neck dissection with adjacent tissue transfer closure. Resection of the posterior scalp and right retroauricular and suboccipital region was consistent with pleomorphic dermal sarcoma, positive for lymphovascular and perineural invasion. There were also 14 lymph nodes containing metastatic pleomorphic dermal sarcoma out of 55 examined lymph nodes. The tumor was staged as T3N2c.

Given the presence of multiple involved lymph nodes, presence of lymphovascular and perineural invasion, and aggressive pleomorphic dermal sarcoma pathology, adjuvant radiation therapy was recommended to reduce the risk of locoregional recurrence, with a recommended dose of 60 Gy delivered in 30 fractions.

### Patient case 3

The third patient is a 48-year-old female with past medical history significant for neurofibromatosis type 1 (NF1), antiphospholipid syndrome, and triple negative invasive breast ductal carcinoma, presenting with malignant peripheral nerve sheath tumor of the posterior occipital scalp. The patient was initially diagnosed with breast cancer after mammographic screening. Further work-up with PET/CT showed persistent moderate to intense FDG activity associated with a cystic and solid occipital scalp mass. Ultrasound-guided core biopsy of the occipital mass 4 months later returned as malignant peripheral nerve sheath tumor (MPNST). Resection of the left posterior neck sarcoma also returned as MPNST, measuring 8 cm in size, with all margins negative for malignancy with the closest margin being 0.2 cm on the deep margin. No extranodal extension or metastases were identified. The tumor was staged as pathologic T3N0.

Given the presence of a large primary tumor as well as close margins, adjuvant radiation therapy was recommended to reduce the risk of locoregional recurrence. Postoperative radiotherapeutic management of soft tissue sarcomas often requires doses of at least 60 Gy [13, 14]. A dose of 60 Gy was planned to the postoperative bed plus a margin created in the superior-inferior direction of the occipital nerves, the likely nerve origin of the MPNST followed by a sequential 6 Gy boost to the postoperative bed with a smaller margin, indicated for the presence of close surgical margins. Given the thin skin located on the posterior scalp, the use of a bolus was needed to ensure appropriate dosing. The fraction size was 2 Gy per fraction [15].

### Patient case 4

The fourth patient is a 39-year-old male with a right posterior scalp melanoma that was initially excised with wide local excision with sentinel node biopsy, with two out of nine sentinel nodes positive for malignancy, followed by a completion lymph node dissection. The patient then received 4 cycles of adjuvant nivolumab for node-positive disease. The patient then developed a biopsy-proven local and nodal recurrence that was resected with lymph node dissection and local tissue rearrangement.

Due to the high-risk nature of his disease, including short interval to local and nodal recurrence and multiple involved lymph nodes, adjuvant radiation therapy was recommended to reduce the risk of locoregional recurrence, with a dose of 48 Gy delivered in 20 fractions targeting the posterior scalp extending from the vertex to the occiput and the involved neck, based on improvement on lymph-node field relapse with adjuvant radiation therapy in the ANZMTG 1.02/TROG 02.01 phase 3, randomized controlled trial [16, 17].

### Patient case 5

The fifth case is a 75-year-old male who presented with a small, pigmented lesion on the left posterior scalp that was initially treated with cryoablation. The patient then developed a local recurrence, which was biopsied with the pathology consistent with desmoplastic melanoma. The patient then underwent wide local excision with sentinel node biopsy and Integra placement. The pathology was consistent with a 5.5 mm, Clark level IV, pure-type desmoplastic melanoma. The tumor was positive for neurotropism. No sentinel nodes were positive for metastatic melanoma.

Given the location on the head and neck, pure desmoplastic melanoma histologic subtype, and presence of neurotropism, adjuvant radiation therapy was recommended to reduce the risk of local recurrence. Desmoplastic melanoma subtypes are relatively rare with a high risk of local recurrence and lower risk of regional or distant metastases. Therefore, adjuvant radiation therapy is commonly advocated to optimal local control for patients with desmoplastic melanoma [18–22]. The patient had limited means of transportation for which a hypofractionated dose treatment plan was recommended. He received a dose of 30 Gy delivered in 5 fractions, a commonly used postoperative radiotherapy regimen for patients with melanoma in the head and neck [23].

### Radiotherapy details

All patients were planned using CT-based simulation and planning. Each patient was immobilized in the supine position on a headrest with a MOLDCARE cushion (QFix) and a ZENTEC IMRT-Reinforced Thermoplastic Mask (CIVCO Medical Solutions). CT simulation was performed on a Philips 16-slice Brilliance large-bore 4-D CT simulator (Philips Healthcare). Intravenous iodine-based contrast was given to aid in vessel and target delineation.

Radiation treatment planning was performed using the Eclipse treatment planning software (Varian Medical Systems). In Eclipse, the MOLDCARE cushion was replaced with a virtual bolus with additional bolus extended to cover the extent of the planning target volume (PTV), as this often exceeded the extent of the MOLDCARE cushion.

**Fig. 1:**
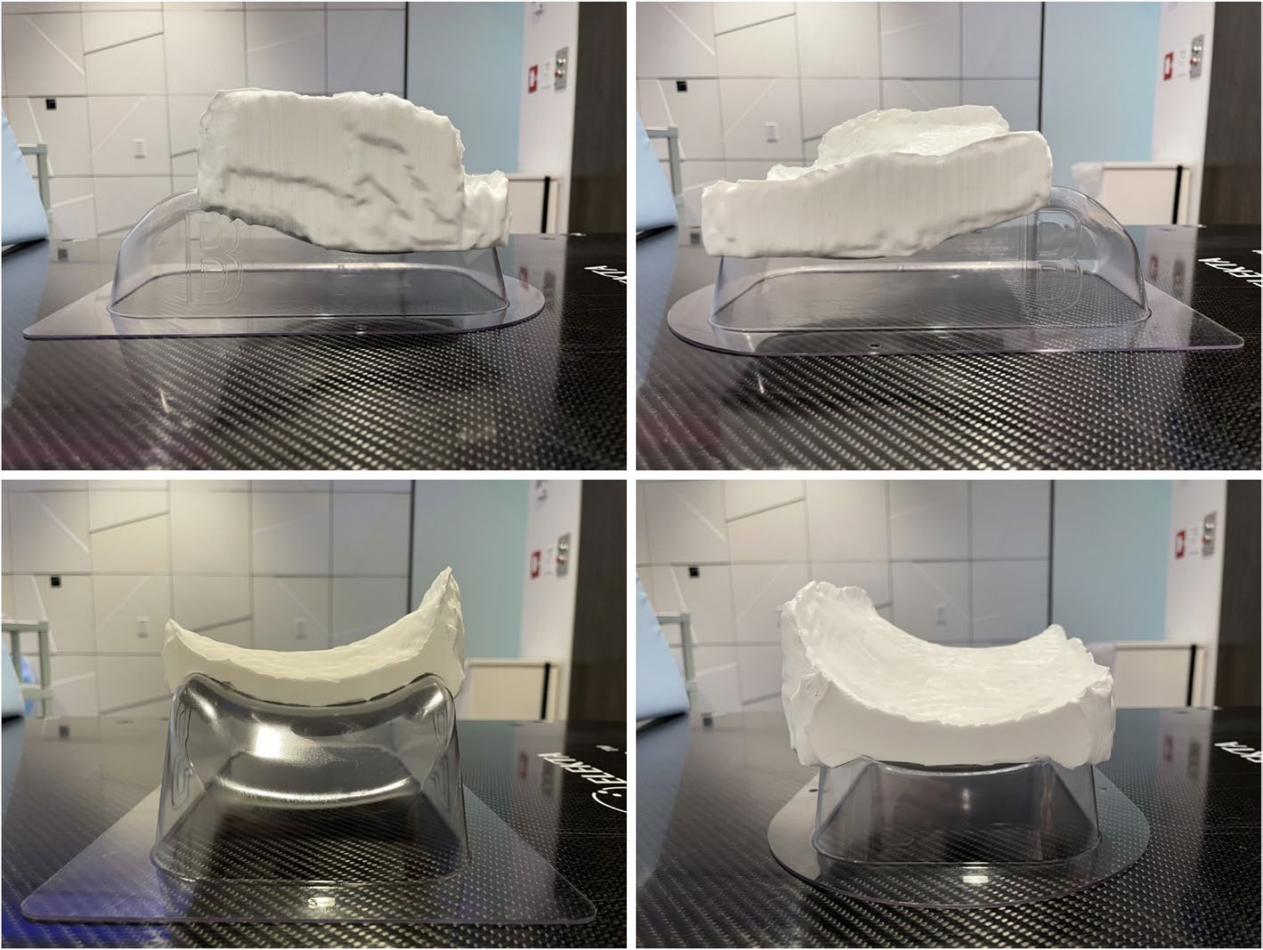
3D printed integrated headrest/bolus on plastic head support. The head support is size B, shown in the left lateral view (upper left), right lateral view (upper right), superior-inferior view (bottom left), and inferior-superior view (bottom right)

Each new integrated bolus/headrest structure was created in Eclipse. The mold was then converted to an STL file using in house software and DICOM structure set [24]. The mold was then printed using polylactic acid on a Makerbot Replicator Z18 (MakerBot Industries, LLC, Brooklyn, NY). The mold was then filled with silicone (Mold Star™ 30; Smooth-On, Inc., Macungie, PA) and allowed to cure for four to eight hours (Fig. 1). Afterwards, the 3D printed integrated bolus/headrest was removed from the mold, tested for shape stability, and confirmed for fitting against the patient.

**Fig. 2:**
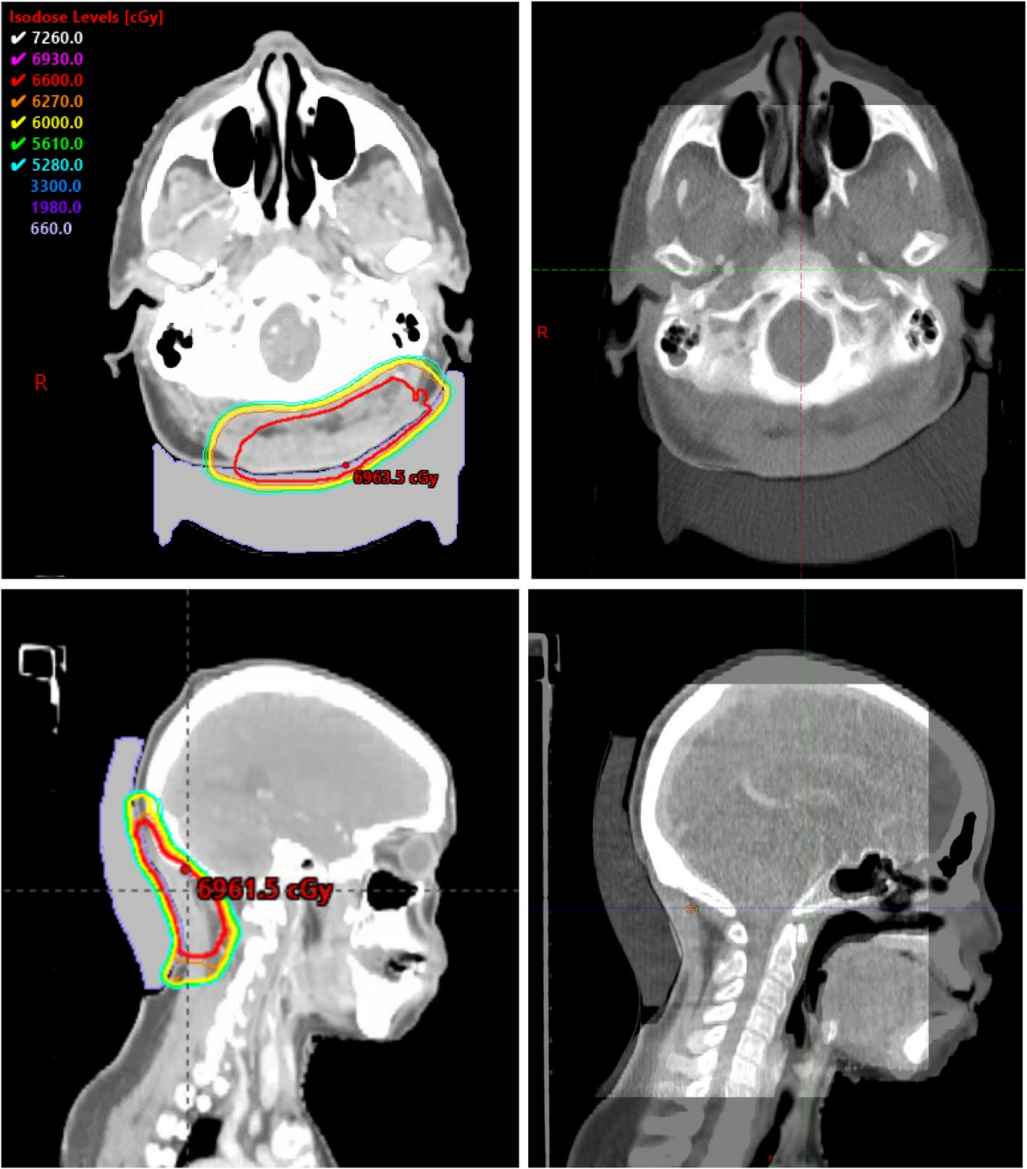
Radiation treatment plan and representative daily cone beam CT (CBCT). The plan summation for the dose distribution of case 3 showing coverage of the posterior scalp with a dose of 60 Gy with a sequential boost of 6 Gy to the surgical bed, with margin (left upper panel). The max point dose is 105.5% of the prescription dose. The upper right panel shows a representative image of the daily CBCT overlaid with the simulation CT, demonstrating consistent daily set up with minimal air gaps between the 3D-printed integrated headrest/bolus and the posterior scalp. The bottom left panel shows the dose distribution in sagittal view and the representative daily CBCT imaging on the bottom right panel in the sagittal view

Patients were set up daily according to their CT simulation specifications, with the integrated 3D printed bolus/headrest used in lieu of the MOLDCARE cushion (Fig. 2). All patients were treated with static intensity modulated radiation therapy or volumetric modulated arc therapy. At the time of the first radiation treatment fraction, optically stimulated luminescent dosimeter (OSLD) was used to measure the dose at the skin surface for validation, as treatment planning systems are typically unreliable for skin dose estimation. Daily cone beam CT was utilized to ensure unchanged daily patient positioning as well as the reproducibility of the treatment setup with the 3D printed integrated bolus/headrest. Grading of all toxicities, including radiation dermatitis, utilized the Common Terminology Criteria for Adverse Events version 5.0.

### Treatment outcomes

The 3D printed integrated bolus/headrest was used for five different patients in their radiotherapeutic management of their malignancy, all located along the posterior scalp. Each radiation therapy set up was verified using daily cone beam CT imaging prior to each treatment, demonstrating consistency and reproducibility. The integrated bolus and headrest conformed to the contour of the patient’s posterior scalp and neck without any significant air gaps. At the beginning of each radiation treatment course, an optically stimulated luminescent dosimeter was used to measure the radiation dose at the level of the skin for verification. The measured dose, as compared to the prescribed dose, ranged from a variation of 2.8 to 5.3%.

Patient 1 received 60 Gy radiation over 30 fractions for treatment of a T3N2b adenosquamous carcinoma in the left parietal scalp. The patient had experienced grade 2 dysgeusia and grade 2 radiation dermatitis on the posterior scalp and left neck by the end of the treatment. There was no evidence of disease at 8 months post treatment.

Patient 2 received 60 Gy radiation over 30 fractions for treatment of a T3N2c pleomorphic dermal sarcoma in the posterior scalp. The patient had experienced grade 1 esophagitis, grade 2 dysgeusia, and grade 2 radiation dermatitis by the end of the treatment. The tumor was well controlled until 3 months post treatment, when FDG avid pulmonary nodules in the bilateral lungs were observed on PET/CT. Biopsy of the pulmonary nodules was consistent with metastatic pleomorphic dermal sarcoma, and the patient underwent radiofrequency ablation of the lung nodules.

Patient 3 received 60 Gy radiation over 30 fractions followed by a 6 Gy sequential boost, for the presence of close margins, for treatment of a T3N0 MPNST in the left posterior scalp. Grade 3 radiation dermatitis on the posterior scalp was observed by the end of the treatment, indicated by the broad presence of moist desquamation. The moist desquamation was managed using Aquaphor and Xeroform gauze. There was no evidence of disease at 3 months post treatment.

Patient 4 received 48 Gy in 20 fractions for a recurrent right posterior scalp melanoma, experiencing grade 2 radiation dermatitis that was well controlled with emollients. There was no evidence of disease recurrence at 3 months post treatment.

Patient 5 received 30 Gy in 5 fractions, with two fractions given per week, for a pT4bN0 left posterior scalp desmoplastic melanoma. The patient experienced grade 2 radiation dermatitis that was well controlled with emollients. He had no evidence of disease recurrence at 3 months post treatment.

## Discussion

Custom 3D-printed bolus creation has been emerging as enormously beneficial in ensuring the delivery of therapeutic doses of radiation to the surface of the skin. However, because this technology is so recent, there is a paucity of studies that study its application to superficial tumors, especially head and neck cancers where it may be difficult to evenly overlay a bolus over the skin. The main advantage of using custom 3D-printed boluses over traditional boluses is ensuring an accurate and individualized fit of the bolus against the irregular surface of each patient’s skin without any air gaps that can affect and alter the ionizing effects of therapeutic radiation, particularly the risk of underdosing the superficial layers of skin due to loss of ionization through air. In particular, this ensures a proper dose distribution, decreases off target radiation, and increases reproducibility of dose delivery [25, 26]. This becomes especially important for patients who are receiving higher doses of radiation with each fraction, i.e., patient 5 who had limited means of transportation that lowered the feasibility of a conventionally fractionated treatment regimen.

In our clinical practice, we have moved away from the use of traditional bolus towards the design and implementation of 3D printed bolus for all scalp malignancies. For example, for patients with skin cancers located on the anterior scalp, which ideally should have a favorable topography and surface for adherent contact of a traditional planar bolus, we still observe significant air gaps between the skin and bolus despite efforts to reduce their development (Supplementary Figure and Table 1). We quantified the amount of air gaps in two patients who we previously treated for anterior scalp cancer, with the 3D bolus offering a significant reduction in air gaps, compared to a traditional bolus (*p*-value = 6.26 × 10^–5^). From experiences such as this, our practice now only treats scalp skin cancers with 3D-printed bolus, reserving traditional boluses for small, flat surfaces or incisions.

With our custom 3D-printed boluses, we observe that dosing is uniform and does not produce excess radiation toxicity in patients with posterior scalp malignancies. We demonstrate that minimal radiation spread out beyond the targeted 60 Gy isodose line (Fig. 2). Our measured doses, as compared to prescribed doses, ranged from a variation of 2.8 to 5.3%. This is similar to variation observed in other studies, in which minimum dose differences between actual and reference doses ranged from 0–5% with 3D printed boluses and ranged from 2–24% with paraffin boluses [27]. Through standard daily image guidance, we ensure that the setup is reproducible and consistent for an area that is difficult to set up, such as the posterior scalp. We also observe that using a custom 3D-printed bolus does not appear to significantly increase radiation toxicity. Multiple studies have observed that in head/neck cancer patients treated with radiation with a classic bolus, around 70% exhibit grade 2 and 20% exhibit grade 3+ radiation dermatitis [28, 29]. Similarly, 4 out of 5 of our patients exhibited grade 2 and 1 out of 5 exhibited grade 3 radiation dermatitis.

We chose to 3D print an integrated bolus and headrest as this would allow for patient comfortability and more uniform dose calculation using our treatment planning software. Using a traditional bolus encounters limitations that either complicate radiation planning or reproducibility. First, the posterior scalp is an irregular surface with high propensity for air gaps with commercially available bolus material. Second, the patient would likely be treated in the prone position to allow for placement of the bolus, which may both decrease patient comfort and reproducible positioning. If the patient was to instead be treated in the supine position with a traditional bolus, the placement of a 0.3 to 1 cm bolus would alter the position of the patient’s head, particularly inducing a neck flexion and roll, which introduces additional variability in daily patient positioning. In addition, this change in anatomy and expected occurrence of air gaps would be difficult to account for in the radiation treatment planning software, which would result in a larger PTV and thus off-target dosing. With our 3D printed integrated bolus/headrest, the radiation planning is seamless since the patient head position in the X, Y, and Z axis as well as roll, pitch, and yaw axis, is more consistent and structurally sturdy without sacrificing patient comfort. There was no change in the patient position that was needed to be accounted.

## Conclusions

Taken together, this report suggests that a 3D printed integrated bolus/headrest is technically feasible and clinically usable for patients with malignancies that involve the posterior scalp, illustrating the novel integration of new technologies in the clinic. Adverse effects observed during treatment were not unexpectedly severe, thus indicating the tolerability of using a bolus/headrest produced by 3D printing. Future studies may expand on continuing to apply and optimize the use of 3D-printed boluses in other scenarios. Overall, in the context of feasibility and consistency, using 3D printing to synthesize customized boluses may be a viable option for complexly placed tumors.

## Supplementary Information

**Additional file 1:** **Supplementary Figure 1.** CT scans of example patients using traditional vs 3D printed boluses. **Supplementary Table 1.** Measurements of maximum air gaps between bolus and skin for patients with anterior scalp malignancies.

## Data Availability

The data generated or analyzed during this study are included in this article, or if absent are available from the corresponding author upon reasonable request.
