# Supplementary material for: 3D printed integrated bolus/headrest for radiation therapy for malignancies involving the posterior scalp and neck
Source: 3D Print Med. 2022 Jul 18;8:22. doi: 10.1186/s41205-022-00152-w (PMC9290275; doi:10.1186/s41205-022-00152-w)
Supplement: Supplementary file 1 — Additional file 1: Supplementary Figure 1. CT scans of example patients using traditional vs 3D printed boluses. Supplementary Table 1. Measurements of maximum air gaps between bolus and skin for patients with anterior scalp malignancies. [file 41205_2022_152_MOESM1_ESM.docx]

Supplementary Figure 1:


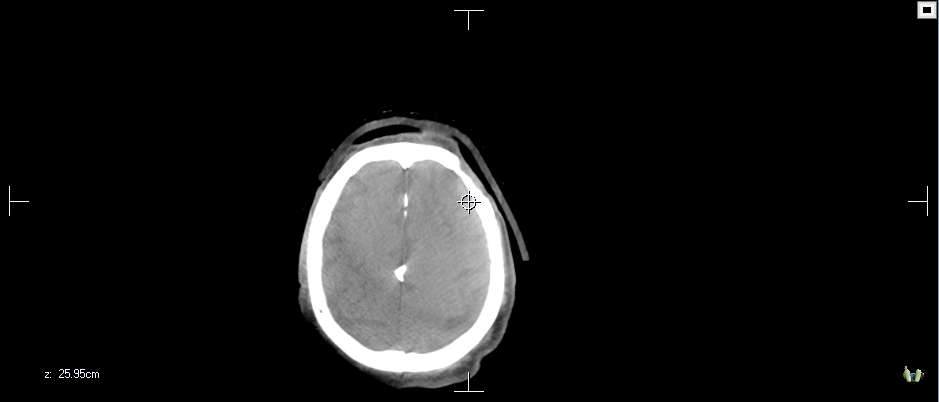


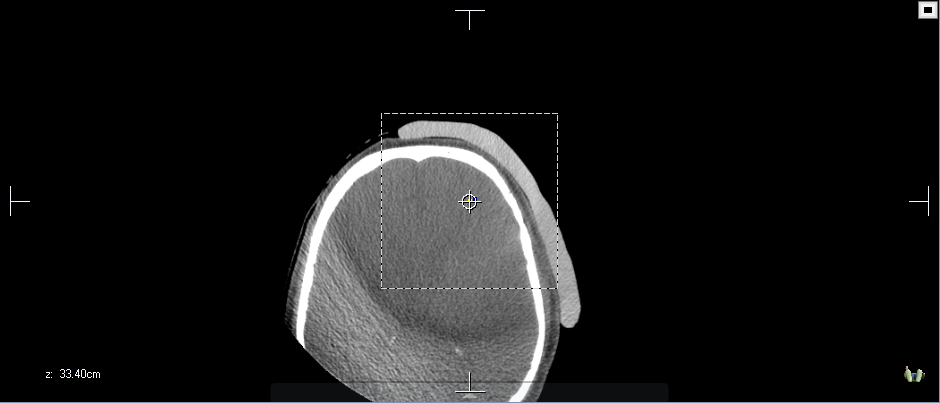


**Supplementary Figure 1. CT scans of example patients using traditional vs 3D printed boluses.** Example of traditional bolus (upper panel) and 3D bolus (lower panel) for anterior scalp radiation therapy.

Supplementary Table 1:

|  | Maximal air gap (in cm) | |
| --- | --- | --- |
|  | Traditional bolus | 3D bolus |
|  | 0.7 | 0.06 |
|  | 0.87 | 0.16 |
|  | 0.81 | 0.2 |
|  | 0.54 | 0.18 |
|  | 0.62 | 0.28 |
| Average | 0.708 | 0.176 |
| SD | 0.134796142 | 0.079246 |
|  |  |  |
| 2 side T-test | 6.25925E-05 |  |

**Supplementary Table 1. Measurements of maximum air gaps between bolus and skin for patients with anterior scalp malignancies.**
